# A Manual of the Diagnosis and Treatment of Diseases of the Eye

**Published:** 1902-12

**Authors:** 


**A Manual of the Diagnosis and Treatment of Diseases of the Eye.** By EDWARD JACKSON, M.D. Pp. 604. Philadelphia and London: W. B. Saunders. 1900.

The chief feature in which this admirable manual differs from other text-books is the very practical way in which each subject is dealt with, and the furnishing of a bibliography at the end of each chapter of the best and most accessible works of reference upon that subject. With so limited a space, the author has naturally had to treat some of the diseases rather briefly. He has, however, found room for a most useful chapter on "Ocular Symptoms in General Disease," and for twenty-two pages on "Remedies and their Applications." The illustrations are not elaborate, but they are numerous, clear, and to the point. The book is thoroughly up to date, and there is no lack of reference to new operations and new drugs. A very full table of contents, and a most complete index, make it a very handy manual for reference, and we can very confidently recommend it to the general practitioner and student of ophthalmology.

